# The Role of Oxidized Cholesterol in Diabetes-Induced Lysosomal Dysfunction in the Brain

**DOI:** 10.1007/s12035-015-9207-1

**Published:** 2015-05-15

**Authors:** Catrina Sims-Robinson, Anna Bakeman, Andrew Rosko, Rebecca Glasser, Eva L. Feldman

**Affiliations:** Department of Neurology, University of Michigan, Ann Arbor, MI 48109 USA; Department of Neurosciences, Medical University of South Carolina, Charleston, SC 29425 USA; Department of Neurology and Neurosurgery, Medical University of South Carolina, 96 Jonathan Lucas Street, 309D2 Clinical Sciences Building, MSC 606, Charleston, SC 29425 USA

**Keywords:** Type 2 diabetes, Brain, Lysosome, Cathepsin D, Cholesterol, Central nervous system

## Abstract

Abnormalities in lysosomal function have been reported in diabetes, aging, and age-related degenerative diseases. These lysosomal abnormalities are an early manifestation of neurodegenerative diseases and often precede the onset of clinical symptoms such as learning and memory deficits; however, the mechanism underlying lysosomal dysfunction is not known. In the current study, we investigated the mechanism underlying lysosomal dysfunction in the cortex and hippocampi, key structures involved in learning and memory, of a type 2 diabetes (T2D) mouse model, the leptin receptor deficient db/db mouse. We demonstrate for the first time that diabetes leads to destabilization of lysosomes as well as alterations in the protein expression, activity, and/or trafficking of two lysosomal enzymes, hexosaminidase A and cathepsin D, in the hippocampus of db/db mice. Pioglitazone, a thiazolidinedione (TZD) commonly used in the treatment of diabetes due to its ability to improve insulin sensitivity and reverse hyperglycemia, was ineffective in reversing the diabetes-induced changes on lysosomal enzymes. Our previous work revealed that pioglitazone does not reverse hypercholesterolemia; thus, we investigated whether cholesterol plays a role in diabetes-induced lysosomal changes. In vitro, cholesterol promoted the destabilization of lysosomes, suggesting that lysosomal-related changes associated with diabetes are due to elevated levels of cholesterol. Since lysosome dysfunction precedes neurodegeneration, cognitive deficits, and Alzheimer’s disease neuropathology, our results may provide a potential mechanism that links diabetes with complications of the central nervous system.

## Introduction

Diabetes mellitus, which currently affects 25.8 million Americans, is a complex metabolic disorder characterized by hyperglycemia. Type 2 diabetes (T2D) accounts for approximately 90–95 % of all diabetes cases and is associated with obesity and hyperinsulinemia. Various complications are associated with diabetes, including retinopathy, nephropathy, neuropathy, and cardiovascular disease [1], and the impact of diabetes on the central nervous system is gaining attention. It is also believed by some that diabetes accelerates brain aging [2, 3]. Aging and age-related diseases may involve abnormalities in the endosomal-lysosomal system, which is an early manifestation of neurodegeneration [4]. Furthermore, lysosomal dysfunction contributes to the accumulation of protein aggregates, a common occurrence in neurodegenerative disorders [5, 6].

Lysosomes are involved in numerous functions, including cell death, exocytosis, endocytosis/phagocytosis, and autophagy. Many of these functions are dependent upon the action of acid hydrolase enzymes within the lysosome that can degrade lipids, carbohydrates, proteins, nucleic acids, or cellular debris. Hexosaminidase A is a lysosomal enzyme that converts GM2 ganglioside to G3M by removing an N-acetyl-glucosamine residue, thereby playing a role in the regulation of insulin sensitivity [7, 8]. In addition, cathepsin D is one of the major lysosomal proteases that contributes to the conversion of proinsulin to insulin in Langerhans cells and rat hepatocytes [9], and it is also involved in the degradation of insulin [10, 11].

Thiazolidinediones (TZDs), a class of drugs known to improve insulin sensitivity, are commonly used for the treatment of diabetes. TZDs are ligands for peroxisome proliferator-activated receptors (PPAR-γ) and improve insulin sensitivity by lowering serum glucose and insulin levels, increasing peripheral glucose uptake, and decreasing triglyceride levels [12, 13]. In fact, studies have demonstrated the beneficial effects of TZDs in neurodegenerative diseases associated with the abnormal accumulation of protein aggregates [14–17]. One such TZD is pioglitazone (Actos), which improves hyperglycemia, reduces hyperinsulinemia, and ameliorates hypertriglyceridemia in a variety of animal models of obesity and insulin resistance [18–23]. Pioglitazone does not improve cholesterol levels in mice (personal communication, [24]).

Hypercholesterolemia is present in 70 % of adults diagnosed with diabetes [25]. In T2D, serum cholesterol is elevated secondary to altered cholesterol synthesis and absorption [26, 27]. When in excess, cholesterol is oxidized by enzymatic or reactive oxygen species (ROS)-mediated pathways. Oxidized cholesterol is increased in T2D [28, 29] and disrupt cellular membranes [30], especially lysosomal membranes [31]. Disrupted lysosomes are incapable of effectively removing ROS-damaged macromolecules [32]. This leads to a feed-forward cycle of damage, where ROS promote the oxidation of cholesterol, resulting in lysosomal injury.

Alterations in lysosomal function in diabetes have been documented in the liver, kidney, heart, saliva, whole brain, and plasma [33–36]. Our previous work in the hippocampi of a well-characterized mouse model of T2D, the db/db mouse, revealed differential expression in genes related to the lysosome [37]. Previous studies have reported alterations in the central nervous system including cognitive impairment and evidence of neurodegeneration in the db/db mouse [38–44]. Therefore, in this study, we characterized lysosomal function in the cortex and hippocampus of the db/db mouse. To determine a potential mechanism underlying lysosomal dysfunction in diabetes, we treated the db/db mice with pioglitazone to ameliorate diabetes and performed subsequent in vitro studies to confirm.

## Materials and Methods

### Animals

Control db+ and T2D db/db mice (BKS.Cg-m +/+ Lepr^db^/J, JAX mice stock no. 000642) were purchased from the Jackson Laboratory (Bar Harbor, ME). Mice were fed a standard rodent chow from Lab Diet (#5053) ad libitum. For pioglitazone studies, db+ and db/db mice were fed a standard diet (5LOD; Research Diets, New Brunswick, NJ) supplemented with or without 112.5 mg of pioglitazone per kg of chow for a final dosage of 15 mg/kg to the mouse beginning at 5 weeks of age. To document the persistence of diabetes, fasting blood glucose levels were measured every 4 weeks by analyzing one drop of tail blood after a 6-h fast using a standard Glucometer (One Touch Ultra, Milpitas, CA). Mice were euthanized at either ∼8 or ∼20 weeks of age.

### Tissue Preparation

The mice were euthanized according to our published protocols with an overdose of sodium pentobarbital, and the tissue was processed as follows per our previously published protocols [45]. For western immunoblotting analyses and enzyme activity assays, the hippocampus and cortex from the dissected brains were prepared as previously described by homogenizing the tissues in tissue protein extraction reagent (Pierce, Rockford, IL) containing a protease inhibitor cocktail (Calbiochem, San Diego, CA). For immunohistochemistry (IHC), mice were perfused with 30 ml of 2 % paraformaldehyde-lysine-periodate, the whole brains were removed and immersed in the same fixative overnight, and the brains were then cryoprotected in PBS (0.1 M, pH 7.2) with 30 % sucrose prior to embedding in OCT compound (Sakura Finetek, Torrance, CA). The brains were then sectioned (20 μm) using a CM1850 cryostat (Leica Microsystems Inc., Bannockburn, IL), mounted onto SuperFrost glass slides (Fisher Scientific, Pittsburgh, PA), and stored at −20 °C until use. For cell fractionation and flow cytometry, the mice were perfused with 15-ml PBS, and the cortex and hippocampus were removed and immediately processed as described below.

### Western Immunoblotting

Western immunoblotting was performed as previously described [45, 46]. Briefly, the tissue lysates were either separated by SDS-PAGE and transferred to a nitrocellulose membrane or used to determine enzyme activity as described below. Tris-buffered saline with Tween-20 supplemented with 5 % milk was used to block the membrane and to dilute the antibodies. Polyclonal antibodies against cathepsin D (Santa Cruz Biotechnology, Inc., Santa Cruz, CA), β-hexosaminidase (ProteinTech Group, Inc., Chicago, IL), and actin (Santa Cruz Biotechnology, Inc., Santa Cruz, CA), as well as appropriate horseradish peroxidase-conjugated secondary antibodies (Santa Cruz Biotechnology, Inc.), were used for western immunoblotting. The signal was visualized using LumiGLO-enhanced chemiluminescence reagent (Cell Signaling Technology, Danvers, MA). Images were captured using the Chemidoc XRS system and analyzed by Quantity One software (Bio-Rad Laboratory, Hercules, CA).

### Enzyme Assays

The activity of β-hexosaminidase A and cathepsin D was measured in 4–5 μg of protein cell lysate from the cortex and hippocampus at 8 and 20 weeks of age (*n* ≥ 5 for each group) in a 96-well plate. To measure β-hexosaminidase A activity, 3.2 mM 4-methylumbelliferyl-6-sulfo-N-acetyl-β-D-glucosaminide (MUGS) potassium salt (Santa Cruz Biotechnology, Santa Cruz, CA) was added to the cell lysate and incubated for 1 h at 37 °C. Next, 2-amino-2-methyl-1-propanol (0.1 M) was added, and fluorescence was read immediately with a 355-nm excitation filter and 460-nm emission filter using a Fluoroskan Ascent II plate reader (LabSystems, Helsinki). Cathepsin D activity was assessed using the cathepsin D activity assay kit (BioVision, Mountain View, CA) according to the manufacturer’s instructions. Fluorescence was read with a 320-nm excitation filter and 460-nm emission filter.

### Cell Fractionation

Dissected cortex and hippocampus were homogenized in homogenization medium (HM: 0.32 M sucrose, 1 mM Na_2_EDTA, 10 mM HEPES; pH 7.0) and centrifuged at 800*g* for 10 min at 4 °C. The supernatant was kept on ice, and the pellet was resuspended in HM and centrifuged again. The supernatants from both centrifugation steps were combined and centrifuged at 20,000*g* for 15 min at 4 °C. The supernatant was centrifuged at 300,000*g* for 2 h at 4 °C to obtain the cytosol, and the pellet was resuspended in HM and layered over a 27 % Percoll solution (Sigma) diluted with Percoll diluent (2.5 M sucrose, 10 mM Na_2_EDTA, 100 mM HEPES; pH 7.0). The sample was centrifuged for 95 min at 20,000*g*. Lysosomes were collected from the layer near the bottom of the gradient and centrifuged for 50 min at 100,000*g*.

### Measurement of Intralysosomal pH

The measurement of intralysosomal pH was performed using flow cytometry with minced cortex and hippocampus (separately) that were trypsinized at 37 °C for 6 min. The tissue was triturated with Leibovitz (L15) media, filtered (70 μm), and centrifuged at 155*g* for 5 min at 4 °C. The pellet was resuspended in PBS. A standard curve was generated in LysoSensor Yellow/Blue DND-160 (2 mM; Invitrogen Molecular Probes) dye-loaded cell suspension using a series of phosphate-citrate buffers containing various mixtures of 300 mM KH_2_PO_4_ and 300 mM citric acid ranging in pH 2–6, supplemented with the inophores nigericin and monensin (Sigma Aldrich; 10 μmol/L) to facilitate the equilibration of intralysosomal pH with the buffer. The cell suspension was allowed to equilibrate for 10 min. The standard curve was generated by exciting at 355 nm and plotting the emission fluorescence ratio (550/21 nm) of DND-160-loaded cells as a function of the actual pH, which was assessed on a standard pH meter. The intralysosomal pH of the lysosomes in the cortex or hippocampus from db+ and db/db mice was calculated by extrapolation from the standard curve.

### Immunohistochemistry

Brain sections were heated on a 55 °C slide warmer for 10 min, hydrated in PBS for 5 min, and permeabilized with PBS containing 0.3 % Triton X-100 and 3 % milk. Sections were incubated in primary antibodies diluted in PBS containing 0.3 % Triton X-100 and 1 % BSA in a humidified chamber overnight at 22 °C. A polyclonal antibody against lysosomal associated membrane protein-1 (LAMP-1; Abcam, Cambridge, MA) was used for IHC. After rinsing with PBS, sections were incubated with the appropriate secondary antibody conjugated with AlexaFluor 594 (Molecular Probes, Eugene, OR) for 1 h at room temperature. Following three rinses with PBS, the sections were incubated for 3 h at room temperature in the dark with 10 μg/ml filipin complex (Sigma, St. Louis, MO). After rinsing with PBS, coverslips were mounted with Prolong anti-fade mounting medium (Molecular Probes, Eugene, OR). Images were captured using a Spot-RT camera (Diagnostic Instruments Inc., Sterling Heights, MI) attached to a Nikon Microphot-FXA microscope.

### Primary Cortical Neuron Experiments

Primary cortical neurons (CN) were prepared as previously described [47]. Briefly, the cortex from E13 B6C3F1/J mice were dissected, dissociated with trypsin, and plated on poly-L-lysine (PLL)-coated tissue culture plates or coverslips. CN were maintained in neurobasal media (Invitrogen, Grand Island, NY) containing 5 mM glucose, supplemented with 1× B27 (without antioxidant; Invitrogen), antibiotics (penicillin, streptomycin, and neomycin; Sigma), 2.5 μg/ml albumin, 10 μg/ml apo-transferrin, 0.1 μg/ml biotin, 15 μg/ml D-galactose, 7 ng/ml progesterone, 16 μg/ml putrescine, 4 ng/ml selenium, 3 ng/ml β-estradiol, 4 ng/ml hydrocortisone, 3 μg/ml catalase, and 2.5 μg/ml superoxide dismutase. CN were cultured for 6 days prior to use, with an addition of fresh media on day 3. CN treatment media (neurobasal media without B27 and antibiotics) was used to carry out the experiments indicated below.

In vitro experiments involved treating primary CN with 25 mM of glucose [47] with or without 5 μM oxidized cholesterol (27-hydroxycholesterol; Medical Isotopes, Inc., Pelham, NH; Prasanthi et al. 2009) for 72 h. Lysosomal destabilization was measured using acridine orange (AO), a lysosomotropic weak base, and metachromatic fluorochrome. Briefly, 5 μg/ml AO in neurobasal medium was incubated with CN on coverslips for 15 min at 37 °C. After the cells were washed and treated as outlined above, microscopic assessment of lysosomes was performed using an Olympus IC-71 inverted confocal microscope and FluoView v4.3 software. Quantitative analysis was performed after staining and treatment of CN in a 96-well clear bottom/black walled plate using a Fluoroskan Ascent FL instrument. When excited by a blue light (450 nm), AO fluoresces red (612-nm wavelength emission) at high lysosomal concentrations and green (520-nm wavelength emission) at low nuclear and cytosolic concentrations. Rupture of AO-loaded lysosomes shows an increase in cytoplasmic diffuse green fluorescence and a decrease in granular red fluorescence [48, 49].

### Statistical Analyses

Data analyses were performed using Prism v6 (GraphPad Software, Inc.). Assumptions about Gaussian distribution of data were made using the D’Agostino and Pearson omnibus normality test. Data not corresponding to a Gaussian distribution were analyzed using an appropriate mathematical transformation, log (*x*). At least 10 mice per group at 8 and 20 weeks of age were used for metabolic studies. For all other measures, at least 5 mice per group were used. *t* test was used in experiments where T2D was compared to the nondiabetic control. For all experiments, *p < 0.05, **p < 0.01, #*p* < 0.001, and Φ*p* < 0.0001 and bar graphs illustrate the mean ± standard error of the mean (SEM).

## Results

### Cortical and Hippocampal Changes in Hexosaminidase A Protein Expression and Activity in T2D

**Fig. 1:**
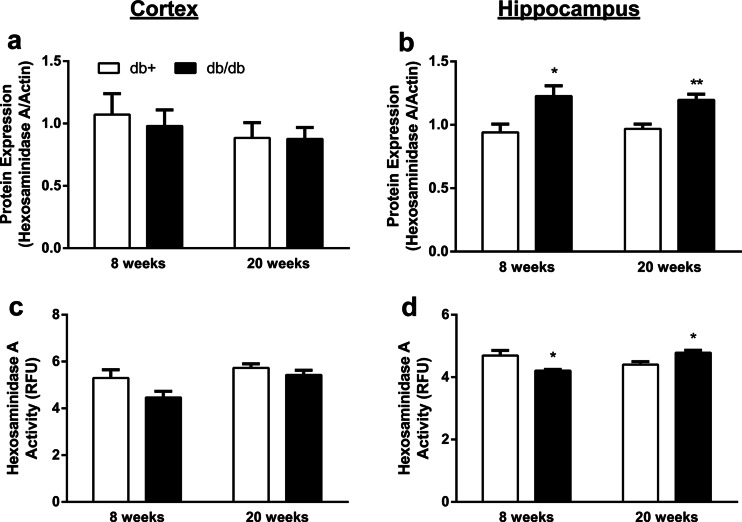
Protein expression and activity of the lysosomal enzyme hexosaminidase A in the brain of T2D mice. **a**, **b** Protein expression and **c**, **d** activity of hexosaminidase A in the cortex (**a**, **c**) and hippocampus (**b**, **d**) of db+ and db/db mice at 8 and 20 weeks of age. Actin was used as the loading control (*p < 0.05 and **p < 0.01 compared with db+; *n* ≥ 6). Activity is expressed in relative fluorescence units (RFU)

We evaluated the lysosomal enzyme β-hexosaminidase A to examine lysosomal function in T2D. While hexosaminidase A protein expression did not change in the cortex, it was significantly increased in the hippocampus by 33 % at 8 weeks and 28 % at 20 weeks in T2D compared to the nondiabetic control (Fig. 1a, b). Likewise, the activity of hexosaminidase A did not change in the cortex; however, it decreased by 10 % in the hippocampus of T2D mice at 8 weeks of age and increased by 8 % in the hippocampus of T2D mice at 20 weeks of age compared to the nondiabetic control (Fig. 1c, d).

### Cortical and Hippocampal Changes in Cathepsin D Protein Expression and Activity in T2D

**Fig. 2:**
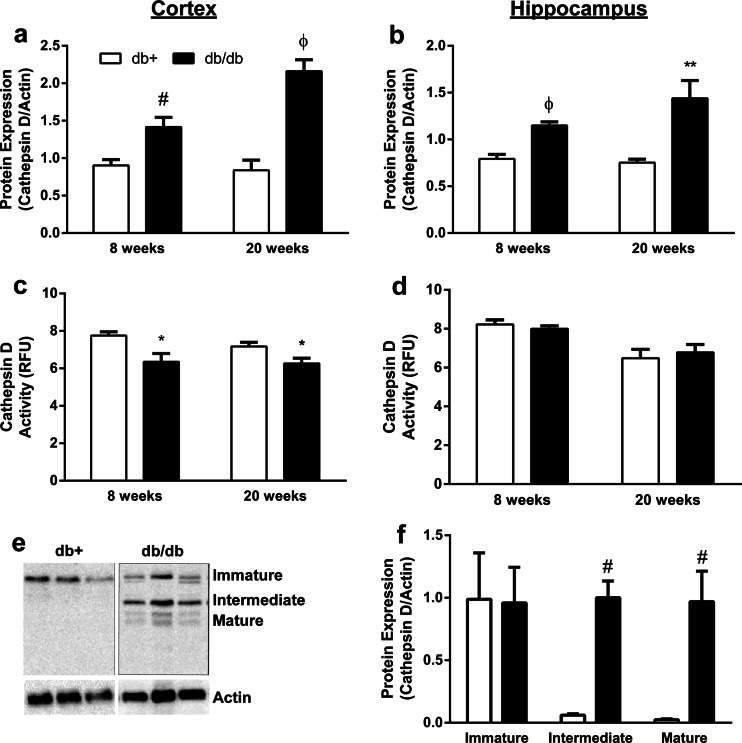
Protein expression and activity of the major lysosomal protease cathepsin D in the brain of T2D mice. **a**, **b** Protein expression and **c**, **d** activity of cathepsin D in the cortex (**a**, **c**) and hippocampus (**b**, **d**) of db+ and db/db mice at 8 and 20 weeks of age. **e** Representative immunoblot and **f** densitometric analysis of the protein expression and subcellular distribution of cathepsin D in the cortex. Actin was used as the loading control (*p < 0.05, **p < 0.01, #*p* < 0.01, and Φ*p* < 0.0001 compared with db+; *n* ≥ 6). Activity is expressed in relative fluorescence units (RFU)

To further examine lysosomal function in T2D, the protein expression and activity of the major lysosomal protease, cathepsin D, were also evaluated. The protein expression of cathepsin D in T2D mice significantly increased by 55 % at 8 weeks and 118 % at 20 weeks in the cortex, and by 46 % at 8 weeks and 64 % at 20 weeks in the hippocampus, compared to nondiabetic control (Fig. 2a, b). This increase in protein expression in the cortex at 20 weeks of age was associated with a 21 % decrease in the activity of cathepsin D in T2D compared to the nondiabetic control, whereas the activity of cathepsin D did not change in the hippocampus (Fig. 2c, d).

The processing of cathepsin D in the Golgi, endosomes, and lysosomes correspond to the 3 major forms—immature, intermediate, and mature, respectively. Alterations in the activity of cathepsin D are associated with the mature form of the protein. Since the protein expression of cathepsin D is significantly increased in the cortex of T2D but the activity is decreased, we assessed the 3 major forms of cathepsin D in the cortex to evaluate whether or not T2D alters the trafficking pattern of cathepsin D. We observed a significant increase in both the intermediate and mature forms of cathepsin D in T2D compared to the nondiabetic control (Fig. 2e, f).

### Lysosomal Membrane Integrity, But Not pH, Is Compromised in T2D

**Fig. 3:**
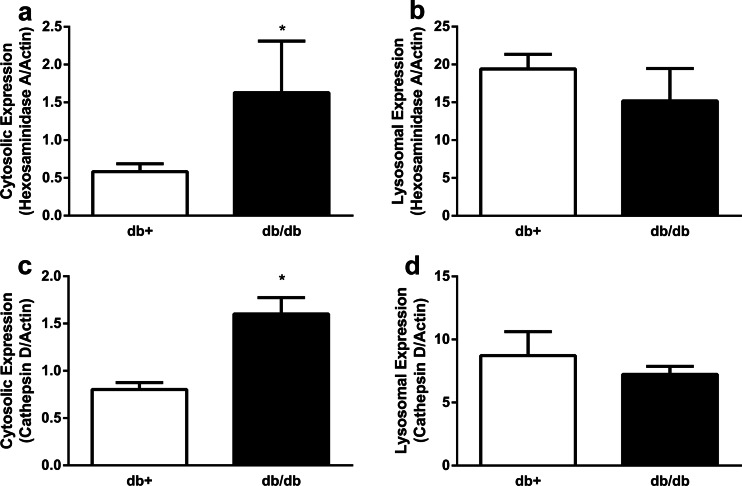
Hexosaminidase A and cathepsin D levels in the hippocampus/cortex of T2D mouse brains. Cell fractionation was utilized to isolate the cytosolic and lysosomal fraction from the hippocampus/cortex of T2D mice at 20 weeks of age. **a**, **b** The levels of hexosaminidase A and **c**, **d** cathepsin D in the cytosolic (**a**, **c**) and the lysosomal fraction (**b**, **d**) from the hippocampus/cortex of db+ and db/db mice. Actin was used as the loading control (*p < 0.05 compared with db+; *n* ≥ 6)

The activity of cathepsin D was expected to parallel the direction of the protein expression of the mature form of cathepsin D; however, we observed a decrease in the activity with an increase in the protein expression of the mature form of cathepsin D in the T2D db/db mouse cortex. This may be due to either a compromised lysosomal membrane with subsequent leakage of the mature form of cathepsin D into the cytosol, or an alteration in intralysosomal pH in the db/db mouse cortex that renders mature cathepsin D inactive. Thus, western immunoblotting following cell fractionation was used to assess the integrity of lysosomal membranes in T2D. The protein expression of both lysosomal enzymes hexsoaminidase A and cathepsin D in the cytosol was increased by at least 100 % in the db/db mouse hippocampus/cortex compared with the nondiabetic control (Fig. 3a, c), whereas the protein expression levels of the lysosomal enzymes were not significantly different in the lysosomal fraction of db/db mice compared with the nondiabetic control (Fig. 3b, d). Flow cytometry was then utilized to assess the potential alterations in the intralysosomal pH in T2D cortex and hippocampus. The average intralysosomal pH in the cortex of the db+ and db/db mice was 4.75 ± 0.87 and 4.71 ± 1.1, respectively (data not shown). Similarly, the average intralysosomal pH in the hippocampus of the db+ and db/db mice was 4.75 ± 0.39 and 4.68 ± 0.39, respectively (data not shown).

### Elevated Levels of Glucose Can Cause Destabilization of the Lysosomal Membrane

**Fig. 4:**
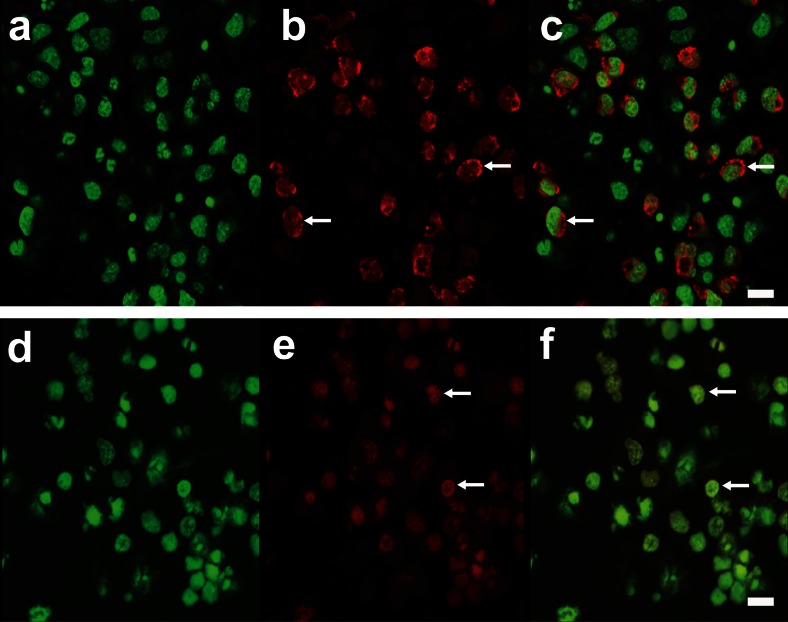
AO staining in CN treated with glucose. **a**–**c** Representative images of control CN and **d**–**f** and CN treated with 25 mM glucose for 72 h. Nuclei are stained; *green and red* puncta represent lysosomes (*white arrows*). *Scale bar* represents 25 μm

To determine a potential mechanism underlying the lysosomal destabilization and leakage associated with T2D, AO staining was used on live primary CN cultures in the presence or absence of 25 mM glucose treatment. An increase in the green cytosolic fluorescence and/or a decrease in red punctate lysosomes are indicative of a loss of membrane integrity. Microscopy of live cells revealed that 25 mM of glucose treatment for 72 h led to the loss of red lysosomal staining (Fig. 4).

### Pioglitazone Improves the Hyperglycemic Phenotype But Not Alterations in Lysosomal Membrane Integrity Associated with T2D

**Fig. 5:**
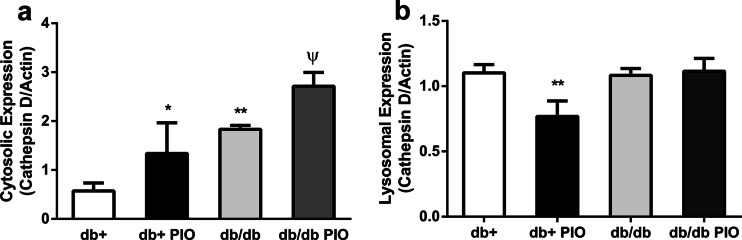
Levels of cathepsin D following treatment with pioglitazone in T2D mice Levels of cathepsin D in the **a** cytosolic fraction and **b** lysosomal fraction of hippocampus and cortex from db+ and db/db mice treated with and without pioglitazone (PIO) at 16 weeks of age. *p < 0.05, **p < 0.01, #*p* < 0.01, and Φ*p* < 0.0001 compared with db+; *n* ≥ 6

Pioglitazone treatment worsens the obesity phenotype of db/db mice and also significantly increases the weight of db+ mice; however, it reverses the elevated levels of blood glucose, glycosylated hemoglobin, and triglycerides in db/db mice back to levels similar to those in control db+ mice (personal communication, [24]). Thus, to determine if pioglitazone can effectively reverse the leakage of lysosomal enzymes into the cytosol, the protein expression levels of hexoaminidase A and cathepsin D were evaluated following pioglitazone treatment in db+ and db/db mice. While pioglitazone did not alter the total protein expression levels of hexosaminidase A or cathepsin D (data not shown), pioglitazone treatment did elevate the levels of cathepsin D in the cytosol following cell fractionation (Fig. 5).

### Elevated Levels of Cholesterol Within Lysosomes in T2D

**Fig. 6:**
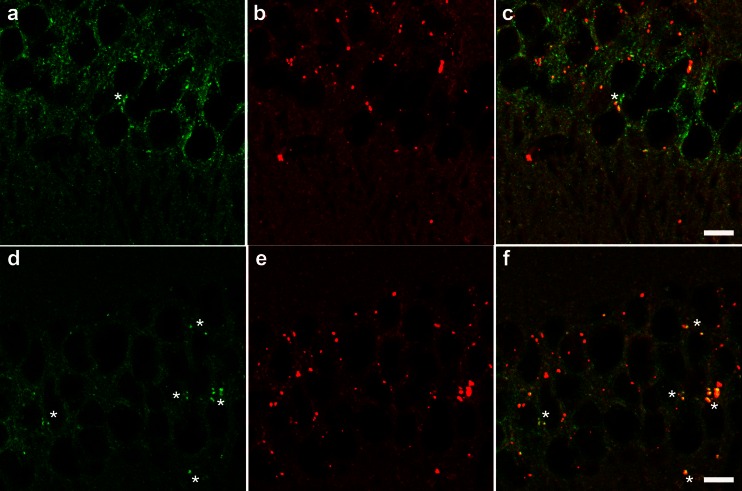
Cholesterol accumulation within lysosomes of T2D mice. Representative images of hippocampus colabeled with filipin (*green*), which stains cholesterol, and LAMP-1 (*red*), which stains lysosomes, in **a**–**c** db+ and **d**–**f** db/db mice. Areas of colocalization (*yellow*) demonstrate cholesterol accumulation within lysosomes (*asterisks*). *Scale bar* represents 10 μm

Fast protein liquid chromatography analyses indicate that pioglitazone treatment does not reverse the levels of cholesterol and promotes an increase in the low density lipoprotein (LDL) cholesterol fraction in db/db mice compared to nondiabetic controls [24]. Thus, to evaluate cholesterol load in the lysosomes of db/db mice, fillipin staining was used. We observed an increase in the colocalization of fillipin and the lysosomal membrane protein LAMP-1 in the cortex and hippocampus of db/db mice compared with db+ control mice (Fig. 6).

### Cholesterol Can Cause Destabilization of the Lysosomal Membrane

**Fig. 7:**
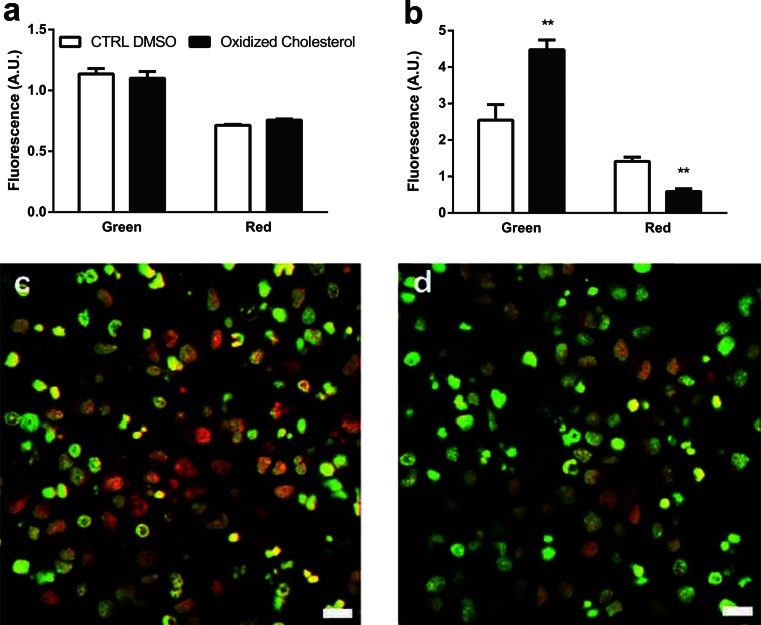
CN treatment with oxidized cholesterol and AO Fluroskan analysis of green and red fluorescence at **a** time 0 and **b** after treatment with control DMSO or oxidized cholesterol for 72 h. Fluorescence is represented by arbitrary units (A.U.). **c**, **d** Images are representative of AO staining following 72 h of **c** control DMSO or **d** oxidized cholesterol treatment, where nuclei are stained *green and red* puncta represent lysosomes. **p < 0.01 compared with control DMSO. *Scale bar* represents 50 μm

To investigate cholesterol as a potential mediator underlying the destabilization and leakage of lysosomes associated with T2D, AO staining was used in live CN treated with and without oxidized cholesterol. Quantitative analysis of the AO staining of live CN revealed that oxidized cholesterol treatment for 72 h led to an increase in green fluorescence and a decrease in the red lysosomal staining (Fig. 7).

## Discussion

In the current study, we investigated the mechanisms underlying alterations in lysosomal function induced by diabetes. Hyperglycemia is a key player in many complications associated with diabetes. Thus, according to the American Diabetes Association, tight glycemic control with either drugs or diet and exercise is the most effective method for preventing diabetic complications. We show for the first time that cholesterol, and not hyperglycemia, may mediate the changes observed in lysosomal function during diabetes in the hippocampus/cortex. Abnormalities in the lysosomal system are early manifestations of neurodegeneration [4]; thus, altered cholesterol metabolism may play a role in diabetes-induced lysosomal changes and neurodegeneration.

The expression of both hexosaminidase A and cathepsin D is upregulated in neurodegenerative diseases [50–53]. Hexosaminidase A removes N-acetyl-glucosamine residue from GM2 ganglioside, converting it to GM3. Obesity leads to a dramatic increase in the protein levels of GM2 in adipose tissue in a mouse model of T2D [54]. Hence, the increase in protein expression observed in the hippocampus of the db/db mouse may be due to obesity. Consistent with our observed increase in hexosaminidase A activity in the db/db mouse hippocampus, T2D patients have increased activity of hexosaminidase A in plasma and serum [36]. Thus, increased activity of hexosaminidase A in plasma and peripheral blood mononuclear cells may have diagnostic value for the detection of the early stages of dementia in Alzheimer’s disease (AD) patients with and without T2D [36].

Cathepsin D is the major lysosomal protease in neurons, contributing to nearly 90 % of protease degradation in the brain [55]. The increase in protein expression we observed in both the cortex and hippocampus is consistent with previous studies in T2D patients reporting that the expression of cathepsin D is increased in serum and leucocytes [56, 9]. On the other hand, the decrease in the activity of cathepsin D we observed is unique to our studies in T2D and may be indicative of abnormalities in the trafficking of cathepsin D to lysosomes. The trafficking of cathepsin D was assessed by evaluating the 3 different isoforms—immature, intermediate, and mature—which correspond to trafficking from the Golgi, endosomes, and lysosomes, respectively [57]. The acidic environment of lysosomes provides a platform for proteolytic cleavage of the intermediate form of cathepsin D to the mature form [57]. Thus, any alterations in the pH of lysosomes may impact the levels of the mature form of the enzyme. Our data indicate that the pH within lysosomes is not altered during T2D. Alternatively, the elevated levels of cathepsin D in the cytosolic fraction in db/db mice suggests that the lysosomal membrane may be compromised. This would explain the elevated protein levels of cathepsin D and the decrease in the activity due to lack of acidic pH in the cytosol. The mechanism underlying this damage to the membrane of lysosomes is not known; however, oxidative stress is associated with leakage of cathepsin D into the cytosol from the lysosome in both in vivo and in vitro models [58, 59].

Hyperglycemia is the key player leading to oxidative stress during T2D. To investigate hyperglycemia as the mechanism underlying lysosomal membrane damage, we exposed CN to hyperglycemic conditions, demonstrating that hyperglycemia causes destabilization of the lysosomal membrane. A previous study demonstrated that hyperglycemia inhibited lysosomal function in macrophages and may contribute to diabetes-associated atherosclerosis [60]. Thus, reversing hyperglycemia may have a profound impact on lysosomal membrane stability.

Pioglitazone is known to improve hyperglycemia, reduce hyperinsulinemia, and ameliorate hypertriglyceridemia in a variety of animal models of obesity and insulin resistance [22, 21, 20, 19, 18]; however, pioglitazone did not reverse the alterations in lysosomal enzymes and in fact lead to a further increase in the levels of cathepsin D in the cytosol. We previously demonstrated that pioglitazone does not improve hypercholesterolemia in db/db mice [24]. In addition, in patients with T2D, serum cholesterol is elevated secondary to altered cholesterol synthesis and absorption [27, 26]. When in excess, cholesterol is oxidized by enzymatic or reactive oxygen species-mediated pathways to generate cholesterol oxides. Cholesterol oxide derivatives, known as oxysterols, are common components of oxidized LDLs, are increased in T2D [29, 28], and disrupt lysosomal membranes [31]. We demonstrate that oxysterols are capable of disrupting lysosomal membranes in primary CN. Thus, it is possible that pioglitazone did not improve the T2D-induced effects on lysosomes due to its inability to reverse hypercholesterolemia in db/db mice.

In summary, it is likely that multiple mechanisms contribute to the alterations in lysosomal enzymes during diabetes. We demonstrate that improving hyperglycemia, insulin resistance, and hypertriglyceridemia alone is not sufficient to reverse diabetes-induced changes in lysosomal membrane stability, although our studies further demonstrate that hypercholesterolemia plays a role in lysosomal membrane destabilization. Future studies will focus on cholesterol as a potential therapeutic target to reverse or prevent the impact of T2D on lysosomal enzymes.
